# Evaluation of the Definitions of “High-Risk” Cutaneous Squamous Cell Carcinoma Using the American Joint Committee on Cancer Staging Criteria and National Comprehensive Cancer Network Guidelines

**DOI:** 10.1155/2014/154340

**Published:** 2014-09-17

**Authors:** Melinda B. Chu, Jordan B. Slutsky, Maulik M. Dhandha, Brandon T. Beal, Eric S. Armbrecht, Ronald J. Walker, Mark A. Varvares, Scott W. Fosko

**Affiliations:** ^1^Department of Dermatology, Saint Louis University, 1402 S. Grand Boulevard, 4th Floor, St. Louis, MO 63104, USA; ^2^Stony Brook School of Medicine, Department of Dermatology, 181 N. Belle Mead Road, Suite 5, East Setauket, NY 11733, USA; ^3^School of Medicine, Saint Louis University, 1402 S. Grand Boulevard, St. Louis, MO 63104, USA; ^4^Center for Outcomes Research, Saint Louis University, 3545 Lafayette Avenue, 4th Floor, St. Louis, MO 63110, USA; ^5^Department of Otolaryngology-Head and Neck Surgery, Saint Louis University, 3635 Vista Avenue, St. Louis, MO 63110, USA

## Abstract

Recent guidelines from the American Joint Committee on Cancer (AJCC) and National Comprehensive Cancer Network (NCCN) have been proposed for the assessment of “high-risk” cutaneous squamous cell carcinomas (cSCCs). Though different in perspective, both guidelines share the common goals of trying to identify “high-risk” cSCCs and improving patient outcomes. Thus, in theory, both definitions should identify a similar proportion of “high-risk” tumors. We sought to evaluate the AJCC and NCCN definitions of “high-risk” cSCCs and to assess their concordance. *Methods*. A retrospective review of head and neck cSCCs seen by an academic dermatology department from July 2010 to November 2011 was performed. *Results*. By AJCC criteria, most tumors (*n* = 211,82.1%) were of Stage 1; 46 tumors (13.9%) were of Stage 2. Almost all were of Stage 2 due to size alone (≥2 cm); one tumor was “upstaged” due to “high-risk features.” Using the NCCN taxonomy, 231 (87%) of tumors were “high-risk.” * Discussion*. This analysis demonstrates discordance between AJCC and NCCN definitions of “high-risk” cSCC. Few cSCCs are of Stage 2 by AJCC criteria, while most are “high-risk” by the NCCN guidelines. While the current guidelines represent significant progress, further studies are needed to generate a unified definition of “high-risk” cSCC to optimize management.

## 1. Introduction

Though it is a rare occurrence, it is well known that prognosis is grim once a cutaneous squamous cell carcinoma (cSCC) has metastasized beyond the skin [1–3]. It would be desirable to be able to identify this small cohort of tumors that are increased risk for metastasis earlier in their presentation to alter treatment approaches and potentially improve outcomes. Recent guidelines from the American Joint Committee on Cancer (AJCC) and National Comprehensive Cancer Network (NCCN) have been proposed to help in the assessment and classification of these “high-risk” cSCCs [4, 5]. Though different in perspective, both guidelines share the common goals of trying to distinguish “high-risk” cSCCs from the bulk of tumors (which are low risk) and trying to optimize patient care. Thus, in theory, both definitions should identify a similar proportion of “high-risk” tumors. In clinical practice, we observed some incongruities between the two definitions. We sought to evaluate the AJCC and NCCN definitions of “high-risk” cSCCs and to assess their concordance.

## 2. Methods

A retrospective chart review of all cSCCs on the head and neck that were initially seen in the Dermatology Department at Saint Louis University Medical Center in July 2010–November 2011 was performed. Data including patient demographics, tumor anatomic location and size, and histologic subtype was documented for all tumors. In addition, the occurrence of any AJCC “high-risk” tumor features detailed in Table 1 (i.e., Breslow depth >2 mm, Clark level >IV, perineural invasion, location on the ear or hair-bearing lip, and poorly differentiated or undifferentiated histology) and the presence of any of the 12 NCCN “high-risk” factors listed in Table 2 (i.e., size by anatomic location, poorly defined borders, recurrence, immunosuppression, site of prior radiation therapy or chronic inflammatory process, rapidly growing tumor, neurological symptoms, moderately or poorly differentiated histology, acantholytic, adenosquamous, or desmoplastic subtypes, depth: ≥2 mm or Clark levels IV, V, and perineural or vascular involvement) were recorded. Data was recorded as documented in the existing medical record. Additional dermatopathologic review of tumor specimens to collect any missing data was not performed.

In the first part of the analysis, tumors were classified according to the AJCC tumor, node, metastasis (TNM) 2010 criteria for cSCC (Table 3) using tumor size and presence of 2 or more “high-risk features” (Table 1). In the second part, the 2012 NCCN guidelines were applied to all cSCC and tumors with 1 of 12 “high-risk” factors were categorized as “high-risk.” Lastly, the proportion of Stage 2 tumors according to AJCC criteria was compared to the proportion of “high-risk” tumors by NCCN guidelines.

## 3. Results

Clinical information was available for 269 cases of the 296 cases identified. The AJCC analysis was based on 257 cases; eyelid tumors were excluded as there is a separate AJCC staging system for eyelid carcinomas (regardless of histologic subtype). The NCCN analysis (classifying “high-risk” versus “low-risk” cSCCs) was based on 265 cases. In 4 tumors, lymph node involvement was uncovered prior to surgery which excluded them from this analysis as tumors with known lymph node involvement are considered in a separate NCCN algorithm rather than the one examined here.

### 3.1. AJCC Analysis

Using the AJCC's tumor (T), node (N), metastasis (M) (TNM) criteria, assignments of Stage 1 and Stage 2 are based on tumor characteristics only (Table 1). Any tumor with a size >2 cm is T2 and thus Stage 2. A tumor that is T1 by size (<2 cm) can be “upstaged” to T2 (and Stage 2) if it possesses ≥2 of the “high-risk” features: depth/invasion >2 mm, Clark level ≥4 mm, perineural invasion, anatomic site on the ear or hair-bearing lip, and poorly differentiated histology. A tumor with an associated positive lymph node is Stage 3 or higher.

**Figure 1:**
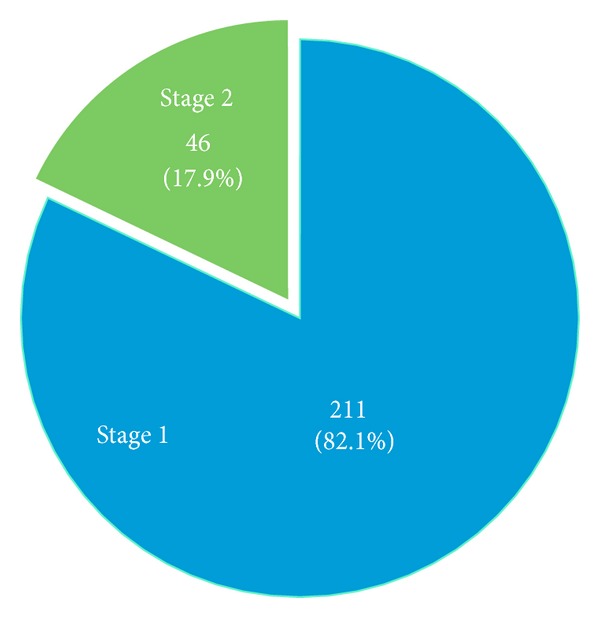
Proportions of Stage 1 versus Stage 2 tumors by AJCC criteria.

**Figure 2:**
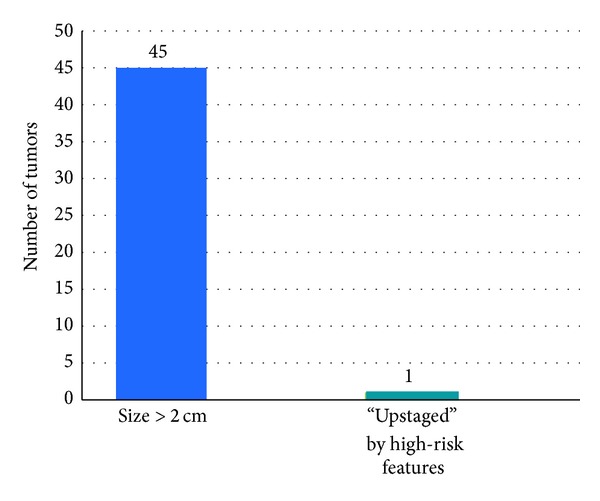
Factor(s) leading to Stage 2 designation by AJCC criteria.

In the AJCC analysis, 257 tumors were included. The majority of cSCCs, 211 (82.1%), were Stage 1; 46 tumors (13.9%) were Stage 2 (Figure 1). Almost all (*n* = 45, 45/46, 98%) were Stage 2 due to size ≥2 cm; only 1 tumor was “upstaged” due to “high-risk features” (Figure 2). This tumor was 1.5 cm in diameter, located on the ear with poorly differentiated histology.

“High-risk” features were not prevalent in our cohort (i.e., perineural invasion (*n* = 12), hair-bearing lip (*n* = 12), ear (*n* = 38)) or almost never recorded. Only 2 tumors had Breslow depth (BD) recorded and Clark level (CL) was never recorded. Of the 2 tumors with BD recorded, only 1 tumor had BD of >2 mm; this tumor met criteria for Stage 2 by size. Histologic subtype was recorded for all 257 tumors; only one tumor had poorly differentiated histology.

### 3.2. NCCN Analysis

The NCCN guidelines are evidence-based algorithms that are intended to guide a clinician through the management of a tumor step-by-step. Unlike the AJCC staging system, within the NCCN guidelines, eyelid carcinomas are grouped together with cSCCs on any anatomic site; thus, eyelid tumors were included in this investigation aimed at classifying tumors as “high-risk” or “low-risk” by NCCN. However, as noted previously tumors with lymph node involvement detected prior to surgery (either by physical exam or by imaging) are considered on a separate different algorithm rather than the one evaluated here and were excluded from this analysis. Taking this into consideration, 265 of 269 tumors were included in the NCCN analysis.

Only 1 of 12 risk factors (Table 2) is required for “high-risk” tumor classification by the NCCN guidelines. Tumors that do not possess any one of these risk factors are considered “low-risk.”

**Figure 3:**
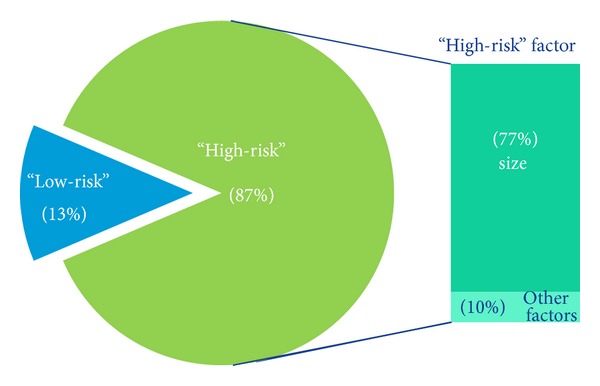
Proportion of “high-risk” versus “low-risk” tumors by NCCN guidelines.

Using NCCN nomenclature, 231 (87%) of tumors were classified as “high-risk,” and 43.2% of tumors had 2 or more NCCN risk factors (Figure 3). Size (≥6 mm on “mask areas of the face” and ≥10 mm on forehead, scalp, cheeks, and neck) was most common risk factor listed. Twenty-seven tumors did not meet “high-risk” by size criteria and were “upstaged” by other clinical risk factors. In this group, the majority of tumors exhibited one “high-risk” factor that led to “upstaging” tumors to “high-risk.” Histologic features were most frequent (moderately/poorly differentiated (*n* = 6) and acantholytic, adenosquamous, or desmoplastic subtypes (*n* = 6)). Clinical designations such as “rapidly growing” (*n* = 4) and “poorly defined borders” (*n* = 6) were also frequently observed.

## 4. Discussion

**Figure 4:**
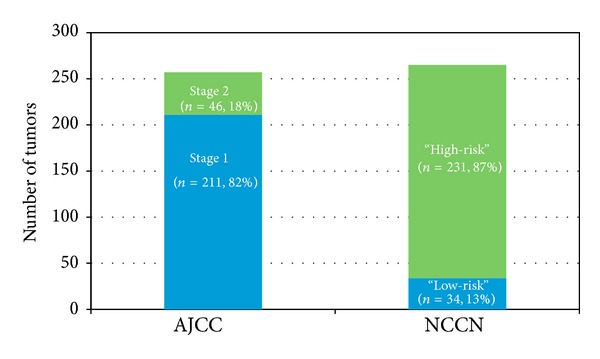
Proportions of “high-risk” and “low-risk” cSCCs by AJCC criteria versus NCCN guidelines.

Our preliminary analysis shows that there is discordance between the AJCC and NCCN definitions of “high-risk” cSCC. Few cSCCs are Stage 2 (14%) by AJCC staging criteria, while most (87%) are “high-risk” by the NCCN guidelines (Figure 4). The AJCC definition of “high-risk” cSCC is narrow, while the NCCN definition is broad.

The purpose of the AJCC staging system is distinct from that of the NCCN treatment guidelines [4, 5]. The goal of the AJCC staging system is to stratify patients with similar outcomes into groups to offer accurate prognostic estimates. AJCC staging is almost entirely based on the “anatomic” characteristics of a primary tumor, using the tumor (T), node (N), and metastases (M) classification [4]. This approach has been criticized as simplistic and in the introduction the authors concede that the restrictive nature of the tumor-based approach has led clinicians to develop other prognostic systems and even led some to conclude that TNM is “obsolete” and “anachronistic” [4]. In fact, most lymphomas and nervous system tumors use alternate systems as the TNM criteria cannot be employed rationally in these cancers [4]. Likewise, it can be awkward to apply the TNM criteria to cSCC.

The omission of established cSCC “high-risk” factors, such as host immunosuppression and tumor recurrence, from the current AJCC staging criteria (AJCC 7th edition) is worthy of discussion. As mentioned above, as a rule the AJCC staging utilizes primary tumor characteristics only and does not permit host factors to be considered. To blindly adhere to these restrictions (which do not allow for the consideration of “immunosuppression” or “recurrence” as “high-risk” factors) appears short-sighted as the exclusion of these important clinical factors may lead to inaccurate prognostic estimates. Like Breuninger et al. [6], the authors believe that a relevant staging system should estimate the risk of metastasis. Hence, when creating a possible new cSCC staging system, Breuninger et al. considered all factors associated with increased metastatic risk and recommended that history of “immunosuppression” should be recorded despite the fact that it was “not a tumor factor” [6].

If the ultimate goal of the AJCC staging system is the “analysis of the care of patients with similar prognosis” [4], then the inclusion of “immunosuppression” and “recurrence” as factors that influence stage determination is apropos and can be considered. The AJCC 7th edition already allows clinically significant “nonanatomic” predictors to be used for staging when the factor's prognostic accuracy has been sufficiently validated [4]. For example, the Féderation Internationale de Gynécologie et d'Obstétrique (FIGO) prognostic score (which is based on 8 factors including patient characteristics/history such as age, antecedent pregnancy, serum human chorionic gonadotropin (hcG) level, and response to chemotherapy) is employed in the staging of gestational trophoblastic tumors [4].

On initial review, there appears to be enough corroborating data to support the use of host “immunosuppression” as a determinant of AJCC stage; its association with increased metastasis has been reported in a number of studies [7–10]. However, a statistically significant association between “immunosuppression” and “metastasis” has not been consistently demonstrated in multivariable analyses. Brantsch et al. [11] found that immunosuppressive status was a significant predictor, while Peat et al., Karia et al., and Jambusaria-Pahlajani et al. did not [12–14]. Jambusaria-Pahlajani et al. acknowledged that although their analysis was based on a large cohort (*n* = 523), the study was underpowered to draw any conclusions on the association between immunosuppression and metastasis [14]. Another possible reason for the exclusion of “immunosuppression” within the AJCC staging criteria may be due to the fact that “immunosuppression” itself is a broad category and may include patients with rheumatoid arthritis on long-term low dose methotrexate, patients with liver or kidney transplants, and patients with chronic lymphocytic leukemia (CLL) or heart or lung transplants on voriconazole. Because cSCC outcomes likely vary widely between these patient cohorts, the AJCC is likely correct to exclude “immunosuppression” as a risk factor required for staging at this time.

While it is still hazy as to what constitutes “immunosuppression,” it is clear that a patient history of “immunosuppression” does figure into a clinician's assessment of metastatic risk. A recent survey of Mohs surgeons showed that more than half of them (55%) thought that “immunosuppression” was an AJCC risk factor [15]. In view of this, we think that even if it is not included as a risk factor required for staging, information regarding “immunosuppression” should be documented on the AJCC staging form. In their critique of the 6th edition of AJCC staging system (AJCC 6th edition), Dinehart and Peterson highlighted “immunosuppression” as a “marquee variable” and recommended that the letter “I” be placed in front of the stage or a separate “H” category (for “host”) be developed to reflect immune status [16]. In their appraisal of the AJCC 7th edition, Warner and Cockerell called the lack of documentation regarding immunosuppressive status “a shortcoming” of the system and advocated for its mandatory documentation [17]. We agree that it is important to document the presence or absence of “immunosuppression” on the cSCC staging form. In order to mirror the staging forms of other cancers, we recommend that a patient's immune status be collected as a “clinically significant prognostic factor,” which allows the data to be recorded even if it is not incorporated into staging calculation. The host factor of “profound immunosuppression” is a “clinically significant prognostic factor” used in the staging of Merkel cell carcinoma (MCC) [18]. The cohorts of patients (e.g., history of HIV/AIDS, CLL, or solid organ transplantation) that are considered to be treated with “profound immunosuppression” in MCC are the same as those that are found to have increased rates of metastasis in cSCC. Therefore, it seems sensible to suggest that “profound immunosuppression” should be added to the AJCC cSCC staging form as a “clinically significant prognostic factor.”

Currently, the TNM classification examines characteristics of primary tumors; as such, “recurrence” by definition cannot be included as a factor for staging. Many analyses aimed at identifying risk factors for cSCC metastasis have found that “recurrent” tumors are more likely to be metastatic than primary tumors [12, 19]. On the other hand, studies focused on evaluating the cSCC AJCC staging system have excluded “recurrence” as a risk factor for metastasis to reflect the standard method of defining metastatic risk based on the attributes of a primary tumor only; consequently, there is no data regarding “recurrent” tumors and its impact on AJCC staging classification. If it is decided that AJCC stage should pertain to primary tumors only, we think that information regarding whether or not a tumor is “recurrent” at the time of treatment should still be recorded on the staging form, either as a “clinically significant prognostic factor” or by placing the letter “r” before the stage as suggested by Dinehart and Peterson [16].

Besides the addition of new factors, we think that the AJCC staging system could be improved by revising the existing T-stage categories. The inadequacy of the AJCC 7th edition cSCC criteria is evidenced by the fact that the T-classification from the European counterpart to the AJCC, the Union for International Cancer Control (UICC), is quite different for cSCC whereas, for most cancers, the AJCC and UICC systems are essentially, if not completely the same [20]. In fact, the UICC T-classification may share more in common with the outdated AJCC 6th edition than the current AJCC 7th edition.

In both the AJCC (6th and 7th editions) and the UICC systems (Table 4), a 2 cm cutoff is used to distinguish between T1 and T2 [4, 20, 21]. When applied to our cohort, the 2 cm size cutoff had important implications. While the majority of tumors were T1 (82%) by AJCC 7th edition, almost all T2 tumors (45 of 46) were T2 due to size alone. In addition, like Breuninger et al. [6], we observed that the expansion of AJCC 7th edition T2 criteria to include tumors of any size that possess two or more “high-risk” features did not significantly change the proportion of T1 and T2 tumors. In our cohort, only 1 of 211 tumors that measured <2 cm had enough risk factors to be “upstaged” to T2.

There were no AJCC 7th edition T3 (tumor with invasion of maxilla, mandible, orbit, or temporal bones) or T4 (tumor with invasion of skeleton (axial or appendicular) or perineural invasion of skull base) tumors in this cohort. The data evaluated here was derived from patients that were initially seen by dermatology, and thus, our study set may be comprised of less aggressive tumors than those examined in other recent studies. However, similar findings to ours (very few T3/T4 tumors) were reported by Breuninger et al. [6] and Jambusaria-Pahlajani et al. [14]. In fact, the lack of T3/T4 tumors in their cohort (4 of 523) led Jambusaria-Pahlajani et al. to conclude that AJCC 7th edition T3 or T4 tumors are so rare that they are unlikely to affect prognostic results [14].

Appreciating that T3/T4 tumors were extremely uncommon and that almost all tumors (98%) were T1/T2, Jambusaria-Pahlajani et al. proposed an “alternative” tumor staging system to improve T-stage stratification [14]. The method, now called the Brigham and Women's Hospital (BWH) system, reportedly provides improved distinctiveness (outcome differences between stages), homogeneity (outcome similarity within stages), and monotonicity (outcome worsening with increasing stage) compared to the AJCC or UICC staging systems [22]. The major differences between BWH system and AJCC 7th edition are as follows: (1) in BWH, size is not the primary determinant of T-stage but one of several prognostic factors (i.e., tumor diameter 2 cm, poorly differentiated histology, perineural invasion 0.1 mm, or tumor invasion beyond fat) that are used in its calculation and (2) T2 is subdivided into T2a and T2b (Table 4). We commend Karia et al. for improving T-stage classification with the BWH system, but think further enhancements can still be made. The factors included in the BWH system were primarily based on the narrow list of AJCC “high-risk” features. Consequently, there may be additional factors (e.g. some factors from the broad list of the NCCN “high-risk” factors) which have not been considered yet and merit inclusion.

As in the BWH system [22], in the system proposed by Peat et al. [12], Breslow depth and Clark level as well as other histopathologic features (e.g., moderate or poor differentiation and/or perineural invasion) were validated by multivariable analyses before inclusion into the staging criteria. Given the strength of the data, it seems reasonable to suggest that BD and CL should be recorded for each tumor. In the present study, we did not collect information on the depth of invasion unless it was recorded on the initial biopsy report. This missing histopathologic data can be considered to be a limitation of our study as the lack of BD or CL (recorded in only 2 of 269 tumors) likely impacted the categorization of tumors as high- or low-risk. It was a deliberate choice of the authors not to perform additional dermatopathologic review as our analysis was intended to demonstrate how staging and classification would apply to data that is routinely recorded during clinical practice. Given the sheer number of SCCs, with estimates ranging from 419,543 to more than 700,000 cases per year in USA, it may not be feasible to require a detailed histopathologic synopsis for cSCC as the one in place for melanoma [23, 24]. In addition, the collection of some histopathologic information like BD or CL which is straightforward to determine for melanomas is challenging or not feasible for cSCCs. For example, collecting precise information on the “true” depth of the tumor is difficult for cSCCs, which may be treated in a variety of ways including Mohs surgery, electrodessication, and curettage or topical 5-fluorouracil, where it may be difficult or impossible to obtain an accurate measurement of tumor depth. However, reliance on a depth reported from the initial biopsy report is also problematic since cSCC diagnosis is usually based on a partial biopsy of the tumor using the shave technique. As the AJCC staging system continues to be refined, it is important that the factors required for staging be not only statistically sound, but also ones that are realistic to collect on a large scale. If the factors are too time consuming to record for every cSCC, classifying tumors with incomplete data may lead to erroneous staging assignment and flawed prognostic estimates.

In contrast to the AJCC staging system which endeavors to stratify patients of similar outcomes and provide prognostic estimates, the NCCN guidelines are intended to direct tumor treatment and management. Using the NCCN classification, almost 90% of the same cohort of tumors analyzed by the AJCC staging system was deemed to be “high-risk,” suggesting that the NCCN's definition of “high-risk” cSCC may be too inclusive. The primary focus in the creation of the NCCN definition of “high-risk” in the 2012 cSCC NCCN guidelines was developing a list of “high-risk” factors for recurrence; identifying risk factors specifically for metastasis (which may overlap with those associated with tumor recurrence) was a secondary concern. Consequently, the “high-risk” criteria used in 2012 NCCN cSCC guidelines may have been kept intentionally broad so as to encompass all factors that have been reported to be associated with a higher rate of recurrence [5].

It is worth mentioning that the list of “high-risk” factors in the 2014 cSCC NCCN guidelines [25] indicates that these factors are “high-risk” for local recurrence OR metastasis; this list is almost exactly the same as the 2012 list of “high-risk” factors which were labeled as “high-risk” for recurrence only [5]. The difference between the two lists is that moderately differentiated histology is no longer considered a “high-risk” factor in the 2014 guidelines. This modification resulted in a negligible change in the percentage of “high-risk” tumors—85.6% (2014) versus 87% (2012)—with only 4 “high-risk” tumors by 2012 NCCN guidelines switching groups when the 2014 NCCN guidelines were applied.

Since only 1 of 12 high-risk factors is needed to for a tumor to be categorized as “high-risk,” tumors with vastly different metastatic potential are all grouped together as “high-risk.” In the current 2014 NCCN guidelines, a tumor that has 4 risk factors (e.g., poorly differentiated 2 cm tumor on the ear invading cartilage with perineural invasion) is considered to be the same as a 6 mm cSCC on the nose, which is considered “high-risk” based on its size and location alone. Even when analyzing tumors that each possess only 1 risk factor (e.g., 5 mm cSCC on the arm in a liver transplant patient maintained on low-dose immunosuppression or a 2 cm well-differentiated tumor on the lateral canthus), the current NCCN guidelines do not distinguish between these two tumors with different levels of metastatic risk.

In summary, our analysis revealed that there is discordance between the AJCC and NCCN definitions of “high-risk” cSCCs. Though the two guidelines are based on different methodologies and serve different purposes, the definitions of “high-risk” cSCC and the cohort they describe should be more similar. When evaluating the AJCC staging system and NCCN cSCC guidelines, it is important to recognize that the cSCC guidelines are still in their infancy as it was not until 2010 that cSCC received a separate set of NCCN guidelines and its own AJCC staging system (in the most recent 7th edition). All in all, the AJCC 7th edition and the NCCN guidelines represent significant progress in the classification and management of cSCCs. Nevertheless, prospective multicenter studies are still needed to determine the role of individual risk factor and the impact of multiple risk factors to generate a unified definition of “high-risk” cSCC in order to develop appropriate treatment guidelines and staging systems that are both practical to implement and accurate in order to optimize the care of our patients.

## Figures and Tables

**Table 1:** AJCC tumor- (T-) staging and “high-risk” features.

Designation	Description

TX	Primary tumor cannot be assessed
T0	No evidence of primary tumor
Tis	Carcinoma in situ
T1	Tumor 2 cm or less in greatest dimension with fewer than 2 high-risk features
T2	Tumor >2 cm in greatest dimension or tumor of any size with two or more high-risk features
T3	Tumor with invasion of maxilla, mandible, orbit, or temporal bone
T4	Tumor with invasion of skeleton (axial or appendicular) or perineural invasion of skull base

High-risk features	

Depth/invasion	>2 mm,
	Clark level ≥ IV, or
	perineural invasion
Anatomic location	Primary site ear
	Primary site hair-bearing lip
Differentiation	Poorly differentiated or undifferentiated

**Table 2:** Abbreviated list of NCCN “high-risk” factors.

NCCN “high-risk” factors^‡^
Area M ≥ 10 mm
Area H ≥ 6 mm
Poorly defined
Recurrence
Immunosuppression
Site of prior RT or chronic inflammatory process
Rapidly growing tumor
Neurologic symptoms
Pathology
Moderately or poorly differentiated histology
Acantholytic, adenosquamous, or desmoplastic subtypes
Depth: ≥2 mm or Clark levels IV, V
Perineural or vascular involvement

Tumor is “high-risk” if ≥1 of 12 risk factors.

M = “medium” risk: forehead, scalp, cheek, neck.

H = “high” risk: “mask areas of the face” central face, ears, periauricular, eyelids, periorbital, nose, temple, and lips.

^‡^Note: The study cohort in this analysis was based on cSCCs from the head and neck only. Risk factors and specifics regarding tumors on Area L (“low” risk anatomic site: trunk and extremities), hands/feet, genitalia are not listed.

**Table 3:** AJCC tumor, node, metastasis (TNM) 2010 cSCC criteria.

Stage	Designation		
	Primary tumor	Regional lymph node	Distant metastasis
0	Tis	N0	M0
I	T1	N0	M0
II	T2	N0	M0
III	T3	N0	M0
	T1	N1	M0
	T2	N1	M0
	T3	N1	M0
IV	T1	N2	M0
	T2	N2	M0
	T3	N2	M0
	T any	N3	M0
	T4	N any	M0
	T any	N any	M1

**Table 4:** Summary of the AJCC 6th edition, AJCC 7th edition, UICC, and BWH tumor- (T-) stage systems.

T-stage	AJCC 6th	AJCC 7th	UICC	T-stage	BWH
T1	Tumor 2 cm or less in greatest dimension	Tumor <2 cm in greatest dimension with fewer than two “high-risk” features∗	Tumor ≤2 cm in largest horizontal size	T1	0 high-risk factors^‡^

T2	Tumor more than 2 cm but not more than 5 cm in greatest dimension	Tumor >2 cm in greatest dimension or tumor any size with two or more “high-risk” features∗	Tumor >2 cm in largest horizontal size	T2a	1 high-risk factor^‡^
				T2b	2-3 high risk factors^‡^

T3	Tumor more than 5 cm in greatest dimension	Tumor with invasion of orbit, maxilla, mandible, or temporal bones	Deep infiltration (skeleton muscle, cartilage, or bone)	T3	≥4 high risk factors^‡^

T4	Tumor invades deep extradermal structures (i.e., cartilage, skeleton muscle, or bone)	Tumor with invasion of skeleton (axial or appendicular) or perineural invasion of skull base	Infiltration of base of skull or vertebra	T4	n/a

*AJCC high-risk factors include >2 mm thickness, Clark level ≥IV, perineural invasion, primary site ear, primary site non-hair-bearing lip, or poorly differentiated histology.

^‡^BWH “high-risk” factors include tumor diameter ≥2 cm, poorly differentiated histology, perineural invasion ≥0.1 mm, or tumor invasion beyond fat (excluding bone invasion which automatically upgrades tumor to BWH stage T3).
